# Understanding rural women's preferences for telephone call engagement with primary health care providers in Nigeria: a discrete choice experiment

**DOI:** 10.1136/bmjgh-2023-013498

**Published:** 2023-12-26

**Authors:** Nasir Umar, Zelee Hill, Joanna Schellenberg, Nuraddeen Umar Sambo, Abdulrahman Shuaibu, Abdulkarim M Aliyu, Kallah Kirpu Kulani, Muhammad U Abdullahi, Ahmed Usman, Hafsat Mohammed, Hajara Adamu, Maryam Ibrahim, Adamu Mohammed, Adama Abdulhamid, Zainab Muhammed, Afodiya Alfayo, Tanya Marchant

**Affiliations:** 1 Department of Disease Control, London School of Hygiene & Tropical Medicine, London, UK; 2 Institute for Global Health, University College London, London, UK; 3 DRMC, Abuja, Nigeria; 4 Office of the Executive Secretary, State Primary Health Care Development Agency, Gombe, Nigeria; 5 Gombe State Primary Health Care Development Agency, Gombe, Nigeria

**Keywords:** health economics, health systems, maternal health, public health

## Abstract

**Background:**

The COVID-19 pandemic has accelerated the use of mobile phones to provide primary health care services and maintain continuity of care. This study aims to understand rural women’s preferences for telephone call engagement with primary health care providers in Nigeria.

**Methods:**

A discrete choice experiment was conducted alongside an action research project that empowered primary health care workers to develop and implement a telephone call intervention to assess and enhance experiences with facility childbirth care. Between January and March 2022, 30 providers from 10 primary health care facilities implemented the choice experiment among rural women who had institutional childbirth to elicit service user preferences for telephone call engagement. The women were asked to express their preferred scenario for telephone call engagement with their primary health care providers. Generalised linear mixed models were used to estimate women’s preferences.

**Results:**

Data for 460 women were available for the discrete choice experiment. The study showed that rural women have preferences for telephone call engagement with primary health care providers. Specifically, women preferred engaging with female to male callers (β=1.665 (95% CI 1.41, 1.93), SE=0.13, p<0.001), preferred call duration under 15 min (β=1.287 (95% CI 0.61, 1.96), SE=0.34, p<0.001) and preferred being notified before the telephone engagement (warm calling) (β=1.828 (95% CI 1.10, 2.56), SE=0.37, p<0.001). Phone credit incentive was also a statistically significant predictor of women’s preferences for engagement. However, neither the availability of scheduling options, the period of the day or the day of the week predicts women’s preferences.

**Conclusions:**

The study highlights the importance of understanding rural women’s preferences for telephone call engagement with healthcare providers in low-income and middle-income countries. These findings can inform the development of mobile phone-based interventions and improve acceptability and broader adoption.

**WHAT IS ALREADY KNOWN ON THIS TOPIC**

- The global spread of mobile phone ownership and the COVID-19 pandemic has accelerated the adoption of mobile phones for primary health care services in low-income and middle-income countries.

**WHAT THIS STUDY ADDS**

- Women preferred engaging with female to male callers, call duration lasting not more than 15 min and being notified before the telephone engagement.
- Women expressed no preference for specific days of the week, times of the day or call scheduling option.

**HOW THIS STUDY MIGHT AFFECT RESEARCH, PRACTICE OR POLICY**

- Healthcare providers and policymakers need to consider women’s preferences for telephone call engagement when designing and implementing telephone-based healthcare interventions in Nigeria as well as similar context in low-income and middle-income countries.
- Further research is needed to explore individual variations in women’s preferences and to identify additional factors that may influence provider-led telephone engagement.

## Introduction

 Mobile phones have been increasingly recognised as an effective means to improve access to and use of health services, particularly among underserved populations.^1^,^3^ Mobile phone ownership has recently increased in low-income and middle-income countries (LMICs), reaching even the most remote areas of sub-Saharan Africa.^2^,^4^ This widespread availability of mobile phones has led to innovative interventions in many aspects of healthcare.^1 3 4^ The COVID-19 pandemic has further accelerated the integration of phone-based engagement into the delivery of healthcare services.^5 6^ One application of mobile phone-based engagement is through telephone calls by healthcare providers to health service users to increase access to care remotely and improve treatment outcomes through early follow-ups, routine symptom monitoring and adherence support.^1^,^4^

For the benefits of telephone call engagement to be realised, service users must have the desire to engage and continue the engagement.^2^ Inappropriate implementation of telephone call engagement may lead to domestic conflict or violence.^7 8^ Additionally, a high rate of respondent attrition and call drop-off has been reported in telephone surveys when the calls are not tailored to respondents’ needs or expectations.^9^ Therefore, it is crucial to ensure that telephone call engagement is implemented in a contextually appropriate manner and that the needs and expectations of service users are taken into consideration to prevent potential harm, maximise the benefits of engagement and improve adoption.^9^,^11^ Consequently, there is a need for more understanding about how service users prefer to engage via telephone, particularly in rural areas.

Qualitative studies have been conducted to explore the feasibility or acceptability of mobile phone use for healthcare services, data collection or engaging service users, including in Bangladesh, Honduras, Brazil, Lebanon, Mali, Peru, South Sudan, Tanzania, Afghanistan, Ethiopia, Kenya, South Africa and Zimbabwe.^12^,^15^ These studies provide insights into the varying levels of acceptability for interventions, research or data collection platforms that rely on mobile phones, taking into account the positive and negative aspects of mobile phone engagement.^12 15^

However, studies on rural women’s preferences for healthcare provider engagement via telephone calls using discrete choice experiment (DCE) in LMICs are more limited. Qualitative research has revealed several aspects of mobile phone interaction or engagement, such as incentives, timing, gender of the caller, mode, scheduling options, solicitation, duration, voice, ease of use and frequency that may impact their acceptability and adoption.^12^,^15^ However, qualitative approaches are limited in quantifying the strength of preference for mobile phone interaction or engagement, a crucial consideration for implementers facing the need to prioritise among numerous aspects of mobile phone interaction or engagement. To overcome this challenge, some studies in high-income countries have employed DCEs to inform programme design and identify potential barriers and facilitators of uptake.^16^,^19^ DCEs are survey-based methods that elicit user preferences and allow the estimation of user values in situations where observable markets are absent or services are provided for free. They can measure the strength of preferences for service attributes, such as mode of delivery, frequency of interaction and provider gender, independently.^20^ Additionally, DCEs can help identify where preferences differ between individuals, particularly when designing interventions that target specific user groups.^11 21^

In this study, we used a DCE in the LMIC setting of northern Nigeria to explore the preferences of service users for telephone engagement with healthcare providers. DCE involves presenting individuals with hypothetical scenarios and asking them to choose their preferred option, enabling researchers to systematically identify the most critical attributes that influence decision-making and design tailored interventions for the target population.^11 22^

Specifically, we focused on rural women’s preferences for attributes of telephone call engagement with primary health care providers about the maternity care they received during their recent facility childbirth. This understanding can help telephone call engagement interventions to be more aligned with the preferences and needs of service users in LMICs, particularly in rural areas.

Ongoing telephone call engagement with women in Nigeria encompasses diverse activities such as data collection, monitoring healthcare satisfaction, issuing reminders and facilitating referrals. The scope of telephone call engagement is anticipated to broaden, encompassing a wider range of primary health care services and ensuring the continuity of care, along with providing supportive supervision. This study aims to understand the preferences of rural women regarding telephone call engagement with primary health care providers in rural Nigeria, with the goal of enhancing the ongoing interaction between healthcare providers and service users via telephone calls, as well as improving the design and implementation of future provider-led telephone call engagement initiatives in Nigeria and other similar settings.

## Methods

### Study context

The study was conducted in Gombe state, Nigeria, with an estimated population of about 3 million as per the last census in 2006.^23^ Among women who gave birth in healthcare facilities, the majority of institutional deliveries occur in public health facilities, while only a small number of women deliver in private health facilities.^24^,^26^ Most of the healthcare workforce in the state consists of lower-cadre health workers, such as community health extension workers (CHEWs), junior CHEWs (JCHEWs) and community health officers (CHOs).^21 27^ In primary health facilities, nurses or midwives are responsible for organising and providing maternal and newborn health (MNH) services with the help of lower-cadre health workers.^21 28^ In the absence of nurses or midwives, these lower-cadre health workers must take full responsibility. Utilisation of MNH services remains low in Gombe state. According to the most recent Demographic and Health Survey, approximately 25% of women did not receive antenatal care, 72% delivered at home and 74% did not undergo postnatal check-ups. Additionally, the maternal and neonatal mortality rates in the state remain alarmingly high, with a rate of 1002 per 100 000 live births for maternal mortality and 42 per 1000 live births for neonatal mortality.^23 25 29^

In Gombe state, healthcare is provided through a network of primary health care facilities managed by local governments and the state primary health care development agency, while secondary health facilities, including general and specialist hospitals, are overseen by the state government. Tertiary healthcare services, such as teaching hospitals and federal medical centres, fall under federal government responsibility. The state’s healthcare system faces a number of challenges of both supply and demand for services. Low demand for healthcare in Gombe state can be attributed to various factors, including economic and geographical barriers, cultural beliefs, religious influences and concerns about care quality. On the supply side, the state faces challenges like workforce shortages and infrastructure limitations. For example, most healthcare workers in Gombe state are lower-cadre personnel, with only a small percentage of medical doctors and nurses/midwives. Many women rely on primary health care facilities staffed by lower-cadre healthcare workers. Bed capacity is limited, with approximately 11–14 beds per 10 000 of the population.^23^,^29^ Outreach programmes remain limited and inconsistent, which highlights the potential for telephone call engagement initiatives to enhance both the demand and supply of healthcare services.

### Study design

The study period, which included training, piloting, DCE data collection and analysis, spans from November 2021 to April 2022. The DCE was conducted between January and March 2022. The study elicited women’s preferences for telephone call engagement with primary health care providers using DCEs, based on different attribute levels presented in table 1.

**Table 1:** Attributes and attribute levels used in the discrete choice experiment on telephone call engagement with primary health care providers

Attributes	Attribute levels and description
Call duration	15 min: telephone call engagement duration lasting not more than 15 min
	45 min: telephone call engagement duration lasting 45 min or more
Period of the week	Weekdays: telephone call engagement occurs Mondays, Tuesdays, Wednesdays, Thursdays or Fridays
	Weekends: telephone call engagement can occur Saturdays or Sundays
Call solicitation	Cold calling: unsolicited calls to individuals who have not expressed any interest
	Warm calling: solicited calls to individuals who have expressed prior interest
Call scheduling	Have the option to schedule calls: women can schedule the date and time of the telephone call engagement
	No option to schedule calls: women cannot schedule the telephone call engagement
Period of the day	Morning/early afternoon: telephone call engagement can occur mornings or early afternoons
	Late afternoon/evening: telephone call engagement can occur late afternoons or evenings
Gender	Female: telephone call engagement to be made by a female
	Male: telephone call engagement to be made by a male
Phone credit	Phone credit: women will receive US$0.5 (200 Nigerian naira) worth of phone credit
	No phone credit: women will not receive any phone credit

#### Identification of attributes and levels

The initial stage of designing the DCE involves selecting the key attributes or characteristics of provider–user telephone engagement that are significant to users.^22 30 31^ To achieve this, we conducted a literature review to identify the attributes of telephone call engagement with primary health care providers, using PubMed and Google data sources. We also examined the reference lists of the identified articles for additional literature. Using the outcomes of the literature review, we conducted a qualitative study that examined the relevance of the identified attributes in the study setting, including call duration, call timing, who should make the call, the method of the call and incentives for call engagement, and established attribute levels by exploring how each attribute could differ. Through the qualitative study involving 50 women, we obtained locally relevant terminology and translations, which enhanced participants’ understanding. The selection of final attributes and levels (table 1) was guided by the need to derive practical and valuable recommendations from the study results, particularly in relation to telephone call engagement with primary health care providers.

#### Experimental design and construction of choice sets

The DCE design involves the combination of attributes and levels into choice alternatives and sets.^22 31^ Due to the number of attributes and levels included in the study, a full factorial design will result in 128 (2^7^) possible combinations of attribute levels, which would have been impractical and burdensome for respondents. Instead, we used a fractional factorial orthogonal main effects design from a design catalogue, which ensured proportional inclusion of levels (level balance) and no correlation between levels of different attributes (orthogonal).^22 32^ This design allowed for a main effects model to be estimated while interactions between attributes were not taken into account.^32^ To reduce respondent burden, we designed 2×8 binary choice sets, and each (eight binary choice sets) was incorporated into a different version of the questionnaire. Additionally, we included a fixed choice set with two alternatives, one of which was strictly dominant over the other, to test for internal validity.^22^ We used binary and forced choices to prevent opt-out options and promote engagement.

**Figure 1:**
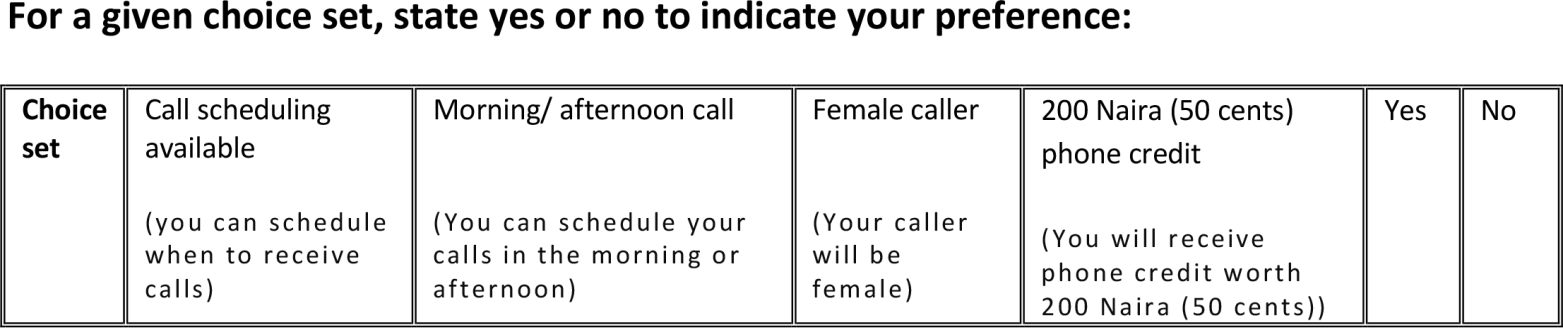
Example of discrete choice experiment choice task as shown to women who had telephone call engagement with primary health care providers.

The content of the choice sets was reviewed and validated with the assistance of a group of healthcare professionals, including doctors, nurses and midwives, who work in Gombe. Subsequently, a pilot test was conducted with 20 women in the study area who share characteristics with the target sample (ie, women who have recently given birth in a primary health care facility). None of the women who participated in the pilot study were included in the main study. The pilot test involved completing the DCE and answering follow-up questions about the exercise, such as the clarity of instructions, the relevance and understanding of the choice sets, and the ease of answering. After the pilot test, minor changes were made, such as adjusting the terminology used to describe attribute levels and choice sets. For example, during the pilot study, participants recommended using the term ‘cellular phone’ instead of ‘mobile phone’ to refer to hand-held devices such as smartphones or basic phones. This was because the word ‘sellula’ is commonly used in the Hausa language (the predominant language in the study setting) to describe these devices. The final DCE tool (figure 1) was incorporated into a larger study questionnaire that also included questions about the participant’s care experience during institutional delivery. The final questionnaire was programmed using CSPro.^33^ The DCE was administered in Hausa language.

#### DCE study sample and data collection

Four hundred sixty rural women who had given birth in the study’s primary health care facilities completed the DCE. Orme^34^ suggests that the minimum sample size required for main effects depends on the number of choice tasks (t), the number of alternatives per task (not including the non-alternative) (a), and the number of analysis cells (for main effects, c is equal to the largest number of levels for any one attribute (c), where n is the number of respondents).^34 35^ Based on the formula n≥500 c/(t×a), an approximate sample of 30 participants would have been sufficient to model our preference data. Lancsar and Louviere also suggest that a sample of 20 respondents per questionnaire is adequate to estimate reliable DCE models.^36^

The questionnaire comprised two sections: the first part included questions about the mothers’ recent experiences with facility childbirth and predischarge counselling, while the second part contained the DCE questions about what they would prefer, reflecting on the information they had just shared. As healthcare providers (male and female) made the calls to women who delivered in their health facilities, no sociodemographic questions were included to ensure the women’s privacy and confidentiality. In addition, the study obtained information on the women delivering in the participating primary health care centres (PHCs) by extracting data from their labour and delivery registers, which were recorded by trained data collection staff on a monthly basis during the study period from November 2021 to April 2022, to understand the characteristics of the women delivering in the study health facilities.

#### Data collection

The study included rural women who gave birth in institutional settings between January and March 2022 at 10 primary health care facilities. These women willingly provided their phone numbers, agreed to follow-up calls and gave their consent to participate. Healthcare providers from the 10 study facilities conducted the choice experiment remotely, making phone calls to the participants. They administered the DCE during these calls, entering the responses into a tablet-based questionnaire designed for the study. The entire DCE process, including the phone interaction and data entry, took approximately 30 min per participant. During the DCE, the women were engaged in discussions to understand their preferred scenarios for telephone call interactions with their primary health care providers. Data collectors and supervisors underwent a comprehensive 5-day training programme. This training covered essential aspects of data collection techniques and the proficient use of study tools. We analysed the DCE data using STATA V.15.

#### Model specification

We analysed the discrete choice data using a random utility model specified within a utility-maximising framework. This framework assumes that when presented with multiple alternatives, a respondent (i=1, …, N) will choose the alternative that yields the highest utility among the available options (j=1, 2, 3, …) at the time of choice.^22^ The respondent’s utility is composed of a deterministic or observable component and a random error component:


(1) $$\begin{array}{l} U_{ij}=v_{ij}+\epsilon_{ij} \end{array}$$

The utility of a respondent, *U_ij_,* is composed of an observable component, *v_ij_,* and a random error term, *ε_ij_,* which has standard statistical properties. Based on equation (1), we model the probability of a respondent selecting telephone call engagement with primary health care providers. We determine the probability of choosing a telephone call engagement by considering the indirect utility function for the respondent *i,* for choice *j* in choice set *s*. We assume that this function is linear, additive and takes the following form:


(2) $$\begin{array}{l} V_{ijs}=X_{ijs}\beta +\epsilon_{ijs} \end{array}$$

The utility derived from a choice is represented by *V_ijs_,* where *X_ijs_β* represents the utility component and *εijs* represents the random component. The vector *X_ijs_* is specified below, where *β_1–7_* represents the design attributes of the choice experiment, and *β_0_* represents the constant.


(3) $$X_{ijs}\beta_{j}=\beta_{0}+\beta_{1}\;call\;duration_{j}+\beta_{2}\;period\;of\;the\;week_{j}+\;\beta_{3}\;call\;solicitation_{j}+\beta_{4}\;call\;scheduling_{j}+\;\beta_{5}\;period\;of\;the\;day_{j}+\beta_{6}\;gender_{j}+\beta_{7}\;phone\;credit_{j}$$

#### Data analysis and model estimation

Two generalised linear mixed models were employed to analyse the data.^37^,^39^ The first model (model 1) included four independent variables: call scheduling (call scheduling, no scheduling), period of day (morning or early afternoon, late afternoon or evening), gender of the caller (female, male) and phone credit incentive (phone credit, no phone credit), as shown in equation (4):


(4) $$\begin{array}{l} logit\left(U_{ij}\right)=\beta_{0}+\beta_{1}\;call\;scheduling_{j}+\beta_{2}\;period\;of\;the\\ day_{j}+\beta_{3}\;gender_{j}+\beta_{4}\;phone\;credit_{j}+b_{ij} \end{array}$$

where *β_1_*, *β_2_*, … *β_4_* are the unknown parameter coefficients of fixed effects, and *b_ij_* as the respondent ID cluster-level random intercepts.

The second model (model 2) also comprised four independent variables: phone credit incentive (phone credit, no phone credit), call solicitation (warm calling, cold calling), period of the week (weekday, weekend) and call duration (15 min, 45 min), as shown in equation (5):


(5) $$\begin{array}{l} logit\left(U_{ij}\right)=\beta_{0}+\beta_{1}\;call\;solicitation_{j}+\beta_{2}\;period\;of\;the\;week_{j}+\\ \beta_{3}\;call\;duration_{j}+\beta_{4}\;phone\;credit_{j}+b_{ij} \end{array}$$

where *β_1_*, *β_2_*, … *β_4_* are the unknown parameter coefficients of fixed effects, and *b_ij_* as the respondent ID cluster-level random intercepts.

Phone credit incentive was a common independent variable in both models, and the dependent variable for both models was women’s preferences for telephone engagement for a given scenario. We presented findings below in line with Strengthening the Reporting of Observational Studies in Epidemiology statement.

#### Patient and public involvement

We fostered participation and engagement among various stakeholders, including women in the community and healthcare providers. We involved the healthcare providers in co-designing and implementing the telephone interview intervention. To ensure the appropriateness and comprehension of the telephone interview protocol, we conducted preliminary consultations with a separate group of women to pretest the protocol. We used their feedback to refine and finalise the telephone interview protocol. We solicited feedback from the participants on the telephone interview process, including perceived difficulty, compatibility and clarity of instructions.

## Results

### Characteristics of women who delivered in the study PHCs

During the study period from November 2021 to April 2022, a total of 1635 women gave birth at the participating PHCs. The age of the women at the time of childbirth ranged from 14 to 48 years, with almost half falling within the 20–29 years age group and a quarter being 19 years or younger. Of the women, 45% had one to three prior childbirths, while approximately 31% had four or more prior childbirths. Additionally, nearly all of the women were Muslim (99%) and married. Nearly half of the childbirths (43%) were assisted by lower-cadre health workers such as community health workers, JCHEWs, CHEWs and CHOs.

### Women’s preferences for telephone engagement with primary health care providers

Out of all the respondents, only three failed the consistency test. The small number of participants failing suggests that the questionnaire was well understood by the majority, and they were able to provide meaningful responses. Consequently, the analysis included the entire dataset of 460 participants, including the data from the three individuals who did not pass the consistency test.

The statistical analysis revealed that both model 1 and model 2 were significant (model 1: Wald Χ^2^(4)=178.54, p<0.001; model 2: Wald Χ^2^(4)=47.59, p<0.0001). This indicates that the independent variables (attributes) were associated with women’s preferences for telephone calls with healthcare providers and accounted for a considerable proportion of the variance in these preferences (table 2).

**Table 2:** Parameter estimates of main effects for women’s preferences for telephone engagement with primary health care providers using GLMM

Attributes and attribute levels	Model 1β (SE)	Model 2β (SE)
Call scheduling: call scheduling option unavailable (ref)		
Call scheduling option available	−0.170 (0.12)	
Period of the day: morning/early afternoon call (ref)		
Late afternoon/evening call	0.084 (0.12)	
Gender: male (ref)		
Female	1.665 (0.13)^a^	
Phone credit: no phone credit (ref)		
Phone credit	0 716 (0.12)^a^	3.363 (0.54)^a^
Call solicitation: cold calling (ref)		
Warm calling		1.828 (0.38)^a^
Call duration: 45 min (ref)		
15 min		1.287 (0.34)^a^
Period of the week: weekends (ref)		
Weekdays		0.017 (0.32)
Number of groups	286	174
Wald Χ^2^(4)	178.54	47.59
Prob>Χ^2^	P<0.001	P<0.001
Random-effects analysis		
SD	1.332	2.592
SE	0.11 (95% CI 1.13 to 1.58)	0.42 (95% CI 1.89 to 3.56)
LR test		
Χbar^2^(01)	162.61^a^	71.83
Prob≥Χbar^2^	P<0.001	P<0.001

β: models’ coefficients.

Significance at 1% (a), 5% (b), 10% (c) levels.

GLMM, generalised linear mixed model; LR, likelihood ratio.

The results indicated that four of the seven independent variables were statistically significant predictors of women’s preferences for telephone call engagement with primary health care providers. Women preferred engaging with female healthcare providers to male healthcare providers (β=1.665 (95% CI 1.41, 1.93), SE=0.13, p<0.001). Women also preferred shorter call duration (β=1.287 (95% CI 0.61, 1.96), SE=0.34, p<0.001) and warm calling to cold calling (β=1.828 (95% CI 1.10, 2.56), SE=0.37, p<0.001). Phone credit incentives were also found to predict women’s preferences for engagement in both models (β=0.716 (95% CI 0.478, 0.95), SE=0.122, p<0.001 and β=1.716 (95% CI 0.10, 2.43), SE=0.37, p<0.001) for models 1 and 2, respectively. However, the availability of scheduling options, the period of the day or the day of the week did not predict women’s preferences.

The random-effects analysis showed that the intercept variance was significant for both model 1 (SD=1.332, SE=0.11 (95% CI 1.13, 1.58)) and model 2 (SD=2.592, SE=0.42 (95% CI 1.89, 3.56)). These findings suggest that the independent variables left considerable variation unexplained in women’s preferences for telephone calls. The likelihood ratio test showed that the generalised linear mixed model was a significant improvement due to the inclusion of the random-effects term for both model 1 (Χbar^2^(01)=162.61, p<0.001) and model 2 (Χbar^2^(01)=71.83, p<0.001), indicating the importance of accounting for individual differences in this analysis.

## Discussion

This study explored the attributes influencing women’s preferences for telephone calls with healthcare providers. The study findings showed that the investigated attributes such as gender, call duration, call solicitation, phone credit incentive, call scheduling, period of the week and period of the day were associated with women’s preferences for telephone calls. Among these attributes, gender, call duration, call solicitation and phone credit incentive influenced women’s preferences for telephone call engagement with primary health care providers. Additionally, the study highlighted substantial individual variation in women’s preferences that could not be explained by the independent variables, emphasising the importance of considering individual differences such as age, education and parity in future research.

The study’s findings indicated that women preferred engaging with female healthcare providers to male healthcare providers through telephone calls. This preference may be because most participants were married and preferred female interviewers to avoid potential marital issues and possibly because the topic was about women’s health. A comparable study using automated voice calls found that female participants preferred a female voice to a male voice.^8^ Another study that investigated the impact of voice characteristics on trust in low-literacy, low-income audiences within a patriarchal context found that the gender or accent of the voice influenced the trust in the information presented, with male participants finding male voice recordings to be more credible than female recordings.^40^ This finding can guide the design of future remote engagement strategies through mobile phones, particularly in patriarchal societies.

The study also revealed that women preferred shorter phone calls, which aligns with similar findings from other studies where participants preferred limited phone interview duration, with 15–30 min being the most cited ideal duration.^14 41 42^ However, some respondents were open to longer conversations if the topic was relevant.^14 41 42^ It is worth noting that service users tend to raise concerns about phone engagement duration more than face-to-face interactions, likely due to the social distance and differing dynamics between the two modes.^14 41^

Moreover, the study showed that phone credit incentives were an important predictor of women’s preferences for engagement. This implies that financial incentives can encourage women’s participation in tele-engagement services. The use of financial incentives has been shown to increase the intention of students, farmers and the general population to adopt new technologies.^8 43^ Our study suggests that financial incentives may increase telephone engagement’s acceptability, motivating people to stay engaged. However, further testing and evaluation are needed to assess its sustainability.

Our findings revealed that women preferred warm calling. Societal norms and attitudes towards receiving calls from unknown numbers may negatively impact the acceptance of telehealth interventions.^8^ Prior research has shown that prenotification letters, emails or Short Message Service can increase engagement rates by legitimising the caller.^8^ Therefore, healthcare providers should prioritise building relationships with their patients or consider sending prenotification messages to increase the acceptability of telephone call engagement.^8^

On the other hand, the study did not find any evidence that women wanted to schedule the calls in advance, nor calls on specific times of the day or days of the week, contrasting previous research that highlighted the ability to schedule calls at convenient times as a factor that increases the acceptability of telephone engagement.^8 44^ Studies have shown that convenience is particularly important for older adults. Giving respondents the option to schedule the call can help mitigate this challenge, especially when they are busy at work or their phone is not charged.^8^ Other studies have reported divergent findings regarding these preferences.^8 41^ The time of day for telephone engagement interventions has been found to influence their acceptance and efficacy.^8^ For instance, previous research has shown that participants preferred the evening between 18:00 and 22:00 for telephone engagement.^43^ Scheduling tele-engagement at an inconvenient time or day resulted in lower participation rates.^43^ Our study also revealed considerable individual variation in women’s preferences for telephone calls that was not explained by the independent variables, indicating the importance of accounting for individual differences in future research.

Finally, ongoing telephone call engagement with women in Gombe state involves various activities such as data collection and monitoring women’s satisfaction with healthcare by the Gombe State Contributory Healthcare Management Agency. Additionally, the scope of telephone call engagement is expanding to encompass broader healthcare service monitoring and supportive supervision. This study was conducted in collaboration with the Gombe State Primary Health Care Development Agency, aiming to enhance ongoing healthcare provider–service user telephone call engagement and to improve the design and implementation of future provider-led telephone call engagement initiatives in the state.

### Strengths and limitations

We adhered to best-practice guidelines for designing the DCE. Yet, the study has some limitations that should be acknowledged. First, the participants were women who had given birth in selected facilities and had provided their contact numbers for the telephone engagement. Consequently, the findings of the study may be more reflective of this particular population and could vary from the preferences of women who give birth at home. Second, the study relied on self-reported preferences, which may not necessarily reflect actual behaviour. Furthermore, sociodemographic data were not collected due to privacy concerns, given that the calls were made by their healthcare providers. Consequently, additional limitation is that no assessment was conducted on additional factors influencing women’s preferences for telephone call engagement, including the investigation of interactions by sociodemographic variables or latent heterogeneity within the study population through latent class analysis. Finally, participants had just completed a telephone engagement, which may introduce social desirability bias in their responses regarding scheduling preferences. However, despite these limitations, the study addresses an important topic regarding women’s preferences for telephone call engagement with primary health care providers. The study employed rigorous statistical analysis, including fixed and random effects, to examine various independent variables’ impact on women’s telephone call engagement preferences. Moreover, the study included a large sample size of women for a DCE study, enhancing the findings’ generalisability to the target population.

## Conclusions

In conclusion, the study provides valuable insights into women’s preferences for telephone call engagement with primary health care providers. The findings indicate that women preferred engaging with female healthcare providers to male healthcare providers, call duration under 15 min, warm calling and phone credit incentives. However, the availability of scheduling options and the time of day were not associated with women’s preferences, and neither was whether the call was on weekdays or weekends. The study has its limitations and strengths, including a large sample size and rigorous statistical analysis. The implications of this study include the need for healthcare providers to consider women’s preferences when conducting telephone calls and the potential benefits of offering phone credit incentives. Future research should explore other factors that may influence women’s preferences for telephone call engagement and consider the impact of demographic factors such as age and race.

## Data Availability

Data are available upon reasonable request.
